# Efficacy of Chloral Hydrate-Hydroxyzine and Chloral Hydrate-Midazolam in Pediatric Magnetic Resonance Imaging Sedation

**Published:** 2014

**Authors:** Razieh FALLAH, Nafiseh FADAVI, Shekofah BEHDAD, Mahmoud FALLAH TAFTI

**Affiliations:** 1 Growth Disorders of Children Research Center, Shahid Sadoughi University of Medical Sciences,Yazd, Iran; 2 Department of Pediatrics, Shahid Sadoughi University of Medical Sciences, Yazd, Iran; 3 Department of Anesthesia and Intensive Care, Shahid Sadoughi University of Medical Sciences,Yazd, Iran; 4 Department of Radiology, Shahid Sadoughi University of Medical Sciences, Yazd, Iran

**Keywords:** Sedation, Children, MRI, Hydroxyzine, chloral hydrate, Midazolam.

## Abstract

**Objective:**

Magnetic resonance imaging (MRI) is a useful diagnostic tool for the evaluation of congenital or acquired brain lesions. But, in all of less than 8-year-old children, pharmacological agents and procedural sedation should be used to induce motionless conditions for imaging studies. The purpose of this study was to compare the efficacy and safety of combination of chloral hydrate-hydroxyzine (CH+H) and chloral hydrate-midazolam (CH+M) in pediatric MRI sedation.

**Materials & Methods:**

In a parallel single-blinded randomized clinical trial, sixty 1-7-year-old children who underwent brain MRI, were randomly assigned to receive chloral hydrate in a minimum dosage of 40 mg/kg in combination with either 2 mg/kg of hydroxyzine or 0.5 mg/kg of midazolam. The primary outcomes were efficacy of adequate sedation (Ramsay sedation score of five) and completion of MRI examination. The secondary outcome was clinical side-effects.

**Results:**

Twenty-eight girls (46.7%) and 32 boys (53.3%) with the mean age of 2.72±1.58 years were studied. Adequate sedation and completion of MRI were achieved in 76.7% of CH+H group. Mild and transient clinical side-effects, such as vomiting of one child in each group and agitation in 2 (6.6 %) children of CH+M group, were also seen. The adverse events were more frequent in CH+M group.

**Conclusion:**

Combinations of chloral hydrate-hydroxyzine and chloral hydrate-midazolam were effective in pediatric MRI sedation; however, chloral hydrate-hydroxyzine was safer.

## Introduction

Magnetic resonance imaging (MRI) is a useful diagnostic tool for evaluation of congenital or acquired brain lesions, dysmyelination or demyelination, gliosis, primary and metastatic brain tumors, cerebral edema, and acute stroke. But, in all of less than 8-year-old children, pharmacological agents and procedural sedation should be used to induce motionless conditions for imaging studies (1), for which different sedation regimens may be used in radiology departments (2,3). 

Schulte-Uentrop and Goepfert (4) in their research concluded that dexmedetomidine may be convenient for MRI sedation of children without cardiac risk. But, the drug is expensive and hardly available in many developing countries such as Iran. 

Chloral hydrate (CH) is a non-opiate, nonbenzodiazepine sedative-hypnotic drug, which has been used for pediatric sedation in a dosage of 40-100 mg/kg for many years (3,5). But, it was not effective in some children, even at maximum dosage, and there are concerns about its long acting duration, obstruction of airway, respiratory depression with intra- and post-procedural oxygen desaturation, sedative effects consistency, and its potential for carcinogenicity, especially at high doses (5-7).

Chloral hydrate at a dose of 40 mg/kg is safer and its combination with antihistamines might decrease chloral hydrate dosage (7,8).

Combination of chloral hydrate and hydroxyzine has been used for sedation of children in dental procedures, and it can decrease the required dosage of chloral hydrate and also cause improvement in safer sleeping of patient and decrease the risk of chloral hydrate related nausea and vomiting (9).

Midazolam is a water-soluble benzodiazepine, which can be used in different routes (oral, intravenous, intramuscular, rectal, sublingual, aerosolized buccal, and intranasal) for sedation induction in children (10,11).

Oral midazolam is a non-parenteral route, which does not cause pain of injection and is used at dosages of 0.5-1 mg/kg in pediatric sedation induction (12).

The purpose of this study was to compare the efficacy and safety of chloral hydrate at minimum dosage in combination with hydroxyzine or midazolam in pediatric MRI sedation induction.

## Materials & Methods

We followed a randomized single-blind study on children who underwent sedation for elective MRI at the Radiology Department of a tertiary-care hospital (Shahid Sadoughi Hospital) in Yazd, Iran from November 2012 to March 2013. 

An Informed consent was taken from patients’ parents before the administration of the drugs, and the study was approved by the Ethics Committee of Shahid Sadoughi University of Medical Sciences, Yazd, Iran.

The sample size was calculated to be 30 children in each group to detect a 20% difference in efficacy between the two groups with type one error (alpha) of 0.05 and 80% power. Eligible participants included children aged 1-7 years, who were in American Society of Anesthesiology (ASA) class 1 (a normally healthy patient) or 2 (a patient with mild systemic disease: mild asthma, controlled diabetes mellitus, etc.) (13). 

Exclusion criteria consisted of presence of gastritis or any other serious systemic diseases, severe systemic reaction, head injury, and receiving a sedative hypnotic agent within the past 48 hours.

The trial used computer generated equal simple randomization by random numbers, and allocation ratio was 1:1 for the two groups. 

Randomization and blinding were performed by an investigator with no clinical involvement in the trial. 

Data collectors, outcome assessors, and data analysts were all kept blinded to the allocation. But, patients and an MRI nurse allocated to the intervention group, were aware of the allocated arm. A pharmacist prepared the drugs, which were given in a suspension of 1 cc/kg. 

The drug combination was delivered by MRI nurses who had certificates in pediatric advanced life support and basic life support, when the participants entered the preparation room. Primary and secondary outcomes were assessed by a pediatric resident who was not informed of the group assignment of drug combination.

The children were randomly assigned to two groups to receive either 40 mg/kg of chloral hydrate and 2 mg/kg of hydroxyzine (Group I) or 40 mg/kg of chloral hydrate and 0.5 mg/kg of midazolam (Group II).

Ramsay sedation scale was used for assessment of the sedation level (14), and it was measured every 10 minutes. The Ramsay sedation scale of five was considered as adequately deep sedation. 

The primary outcomes were efficacy in adequate deep sedation and completion of MRI examination.

The secondary outcomes included clinical side-effects, serious adverse events (hypotension, hypoxia and cyanosis, severe vomiting, intractable irritability and agitation, apnea, laryngospasm, and bradycardia), time from administration of the drug combination to adequate sedation, caregiver’s satisfaction on a likert scale of 1-5 (1= completely unsatisfied; 2= partially unsatisfied; 3= partially satisfied; 4= satisfied; and 5=completely satisfied), and total stay time in MRI center. 

Respiratory depression requiring assisted ventilation, oxygen saturation of less than 90%, or a 25% or greater decrease in pre sedation mean arterial blood pressure were considered as serious side-effects. Failure to achieve adequate sedation (patient’s wakening or movement, interfering with completion of MRI examination, inadequate sedation, and need for administration of other sedatives) and procedure abortion due to serious adverse events, were considered as failure of sedation regimen.

The developmental status of the patient was assessed by a pediatric neurologist based on Denver II Developmental screening test (15).

The strength of the MRI machine of our hospital was 1.5 Tesla, and it had been manufactured by Siemens in 2010. 

Non-contrast brain MRI of these children was interpreted by a pediatric neurologist and a radiologist using a threepoint scale: 1= good quality and no motion, 2= reportable and minor movement, and 3= non reportable and major movement.

The data were analyzed using SPSS statistical software (version 17). Chi-square and Fisher’s exact tests were used for data analysis of qualitative variables, and mean values were compared by independent t-test. 

Kaplan–Meier survival analysis was used to calculate probability of adequate sedation during the observation period. The differences were considered significant at p<0.05.

This study was registered in Iranian clinical trials with registration number IRCT201302092639N10.

## Results

The design and conduct of this trial was straightforward, and we did not have any losses to follow-up or exclusions. 

Twenty-eight girls (46.7%) and 32 boys (53.3%) with the mean age of 2.72±1.58 years were evaluated. No statistically significant differences were observed between the two groups in terms of mean of age, mean of weight, sex distribution, developmental status, and age group of children in two groups (Table 1). Adequate deep sedation (Ramsay sedation score of five) and completion of MRI examination was achieved in 23 (76.7%) children in chloral hydrate-hydroxyzine group [95% confidence interval (CI): 0.61-0.92] and in 22 (73.7%) children in chloral hydrate-midazolam group (95% CI: 0.58-0.89), and the statistical analysis showed that the efficacy of both drugs combination in the sedation induction was not statistically different (p=0.76).

Also, the quality of MRI was not significantly different between the two groups (Table 1).

Table 2 shows comparison of the mean of acquired Ramsay sedation score, time between drugs taking and reaching the Ramsey score of five, caregiver’s satisfaction scale, and total stay time in radiology department which indicates that in chloral hydrate-hydroxyzine group, parents waited less in the radiology department.

Comparison of completion of MRI examination in both groups based on developmental status and age group is shown in Table 3, which indicates that the efficacy of both drugs combination in sedation induction was not statistically different in children with and without developmental delay, in infants (less than two years old), and also in children.

**Fig 1:**
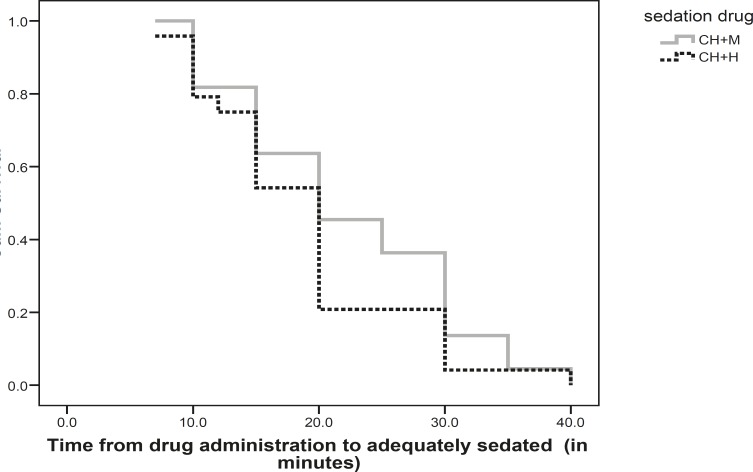
Probability of being adequately sedated vs. time after taking the drugs by Kaplan–Meier plots

Probability of being adequately sedated vs. time after taking the drugs is shown in Kaplan–Meier plots in Fig.1, which indicates that Ramsay sedation score of five was obtained in all children who achieved adequate sedation 40 minutes after taking the drugs combination. No serious adverse events were seen in the two groups. Mild and transient clinical side-effects were seen, such as vomiting in one child in each group and agitation in 2 (6.6%) children of CH+M group. The adverse events were more frequent in CH+M group (p=0.04).

## Discussion

Various drugs have been used for pediatric MRI sedation. The Results of this randomized clinical trial showed that combination of chloral hydrate at minimum dosage and hydroxyzine or midazolam were equally effective in children who underwent MRI.

In another Iranian study, adequate sedation and completion of CT scan examination were achieved in 76.7% of children who aged 1-10 years and received 100 mg/kg chloral hydrate orally (16).

In Mason et al.’s study, efficacy of oral chloral hydrate at a dosage of 50 mg/kg and oral pentobarbital were equal in MRI sedation of younger than 1-year-old infants; 

however, side-effects frequency was significantly lower in pentobarbital group (17).

However, Schulte-Uentrop and Goepfert concluded that in sedation induction for MRI, chloral hydrate, pentobarbital, and midazolam are not proper and dexmedetomidine may be a more effective drug in sedation induction in children without cardiac risk, and if anesthesiologists or pediatric intensivists are present, propofol can be used and general anesthesia is a preferred technique in preterm or small children (4).

In a study in Iowa, USA, a combination of 25 mg/kg chloral hydrate, 1 mg/kg hydroxyzine, and 1 mg/kg meperidine was more effective than 0.65 mg/kg of oral midazolam in sedation for dental procedures (18).

In a study in Miami, USA, efficacy of chloral hydrate, combination of chloral hydrate and diphenhydramine, chloral hydrate-hydroxyzine hydrochloride combination and midazolam alone were compared in case of sedation induction for echocardiography. In chloral hydrate group, children fell asleep the most quickly and chloral hydrate and diphenhydramine group had the most prolonged sedations (19).

In a study in Mexico City, combination of 70 mg/kg chloral hydrate and 2 mg/kg hydroxyzine in comparison with 70 mg/kg chloral hydrate alone caused a significantly more decrease in crying and movement within 45-60 minutes after a rubber dam insertion. But, in both groups, overall behavior was not different during the dental procedures (20).

In a study in Mexico, a combination of 0.50 mg/kg midazolam and 1.5 mg/kg hydroxyzine or 50 mg/kg chloral hydrate and 1.5 mg/kg hydroxyzine were more effective than 2 mg/kg hydroxyzine alone in sedation induction for dental procedures (21).

In the present study, the efficacy of both combination of the drugs in sedation induction were not statistically different in infants (less than two years old) and also in children. But, in a Korean study, chloral hydrate was more effective in MRI sedation of younger than 18-month-old children (22).

In our study, the efficacy of both combinations of the drugs in sedation induction for MRI was not statistically different in children with and without developmental delay, which is in agreement with a study in the USA (23).

Possible explanations for these discrepancies include differences in age, drugs combination and dosage, race, sample size, type of procedure, etc.

In the present study, both sedation regimens were safe and no serious clinical adverse events were seen in the two groups. But, in Fávero et al.’s study, respiratory complications occurred in two of 41 children who received 50 mg/kg of chloral hydrate (24) and in Heistein et al.’s study in Texas, serious side-effects such as apnea occurred in 0.3%, airway obstruction in 1.4%, hypoxia in 5.9%, hypercapnia in 6.6%, and hypotension in 0.4% of children who were sedated with chloral hydrate for echocardiography (25).

**Table 1:** Comparison of Some Characteristics of Children between the Two Groups

Group		Chloral hydrate and hydroxyzine	Chloral hydrate and midazolam	p-value
Data				
Age in year (mean±SD)		2.93±1.56	2.51±1.59	0.3
Weight in kg (mean±SD)		12.68±4.11	12.45±3.24	0.3
Sex	Female	13	15	0.6
	Male	17	15	
Developmental status	Normal	14	17	0.4
	Delay	16	13	
Age group	< 2 years	10	14	0.2
	≥ 2 years	20	16	
MRI quality	No motion	18	19	0.5
	Minor movement	4	2	

In this research, achieving sleep in majority of children who were adequately sedated with the two sedation regimens appeared up to 40 minutes after administration of the drugs combination, therefore, administration of these sedative drugs in 40 minutes before the procedure could be more effective.

**Table2:** Comparison of Mean of Sedation Parameter Variables between the Two Groups

Group	Chloral hydrate and hydroxyzine	Chloral hydrate and midazolam	p-value
Data			
Acquired Ramsay sedation score	5.07± 1.23	4.71± 1.62	0.3
Time between drugs taking and reaching Ramsey score of five (in minute)	18.91± 8.15	22.27± 9.22	0.2
Time after taking the drug to completing MRI examination (in minute)	33.95± 12.06	39.18± 11.21	0.1
Caregiver’s satisfaction scale	3.81±1.27	3.37± 1.41	0.2
Total stay time in radiology department (in minute)	69.1± 22.49	81.81± 23.24	0.03

**Table 3:** Comparison of Success in Completing MRI Examination in Both Groups Based On Developmental Status and Age Group

Success in completing MRI			Yes	No	P-value
Data					
Developmental status	Normal	CH+H	10	4	0.6
		CH+M	12	5	
	Delay	CH+H	13	3	0.6
		CH+M	10	3	
Age group	<2 years	CH+H	8	2	0.5
		CH+M	11	4	
	≥ 2 years	CH+H	15	5	0.6
		CH+M	11	4	

CH+H: Chloral hydrate and hydroxyzine

CH+M: Chloral hydrate and midazolam

**In conclusion**, results of the present study showed that a combination of chloral hydrate at minimum dosage and hydroxyzine or midazolam are equally effective in pediatric MRI sedation. But, a combination of chloral hydrate and hydroxyzine is safer.

## References

[1] Lehman RK, Schor NF, Kliegman RM, Stanton BF, Schor NF, St. Geme JW, Behrman RE (2011). Neurologic Evaluation. Nelson Textbook of Pediatrics.

[2] Sahyoun C, Krauss B (2012). Clinical implications of pharmacokinetics and pharmacodynamics of procedural sedation agents in children. Curr Opin Pediatr.

[3] Mason KP, Prescilla R, Fontaine PJ, Zurakowski D (2011). Pediatric CT sedation: comparison of dexmedetomidine and pentobarbital. AJR Am J Roentgenol.

[4] Schulte-Uentrop L, Goepfert MS (2010). Anaesthesia or sedation for MRI in children. Curr Opin Anaesthesiol.

[5] Freeman JM (2001). The risks of sedation for electroencephalograms: data at last. Pediatrics.

[6] Cortellazzi P, Lamperti M, Minati L, Falcone C, Pantaleoni C, Caldiroli D (2007). Sedation of neurologically impaired children undergoing MRI: a sequential approach. Paediatr Anaesth.

[7] Haselkorn T, Whittemore AS, Udaltsova N, Friedman GD (2006). Short-term chloral hydrate administration and cancer in humans. Drug Saf.

[8] Costa LR, Costa PS, Brasileiro SV, Bendo CB, Viegas CM, Paiva SM (2012). Post-Discharge Adverse Events following Pediatric Sedation with High Doses of Oral Medication. J Pediatr.

[9] da Costa LR, da Costa PS, Lima AR (2007). A randomized double-blinded trial of chloral hydrate with or without hydroxyzine versus placebo for pediatric dental sedation. Braz Dent J.

[10] Klein EJ, Brown JC, Kobayashi A, Osincup D, Seidel K (2011). A randomized clinical trial comparing oral, aerosolized intranasal, and aerosolized buccal midazolam. Ann Emerg Med.

[11] Johnson E, Briskie D, Majewski R, Edwards S, Reynolds P (2010). The physiologic and behavioral effects of oral and intranasal midazolam in pediatric dental patients. Pediatr Dent.

[12] Wetzel RC, Kliegman RM, Stanton BF, Schor NF, St. Geme JW, Behrman RE (2011). Anesthesia, Perioperative Care, and Sedation. Nelson Textbook of Pediatrics.

[13] Cote CJ, Wilson S (2006). Guidelines for monitoring and management of pediatric patients during and after sedation for diagnostic and therapeutic procedures: an update. Pediatrics.

[14] Ramsay MA, Savege TM, Simpson BR, Goodwin R (1974). Controlled sedation with alphaxalone-alphadolone. Br Med J.

[15] Fallah R, Jalili Sh, Golestan M, Akhavan Karbasi S, Jarahzadeh MH (2013). Efficacy of chloral hydrate and promethazine for sedation during electroencephalography in children; a randomised clinical trial. Iran J Pediatr.

[16] Fallah R, Nakhaei MH, Behdad S, Moghaddam RN, Shamszadeh A (2013). Oral chloral hydrate vs. intranasal midazolam for sedation during computerized tomography. Indian Pediatr.

[17] Mason KP, Sanborn P, Zurakowski D, Karian VE, Connor L, Fontaine PJ (2004). Superiority of pentobarbital versus chloral hydrate for sedation in infants during imaging. Radiology.

[18] Chowdhury J, Vargas KG (2005). Comparison of chloral hydrate, meperidine, and hydroxyzine to midazolam regimens for oral sedation of pediatric dental patients. Pediatr Dent.

[19] Roach CL, Husain N, Zabinsky J, Welch E, Garg R (2010). Moderate sedation for echocardiography of preschoolers. Pediatr Cardiol.

[20] Avalos-Arenas V, Moyao-García D, Nava-Ocampo AA, Zayas-Carranza RE, Fragoso-Ríos R (1998). Is chloral hydrate/ hydroxyzine a good option for paediatric dental outpatient sedation?. Curr Med Res Opin.

[21] Torres-Pérez J, Tapia-García I, Rosales-Berber MA, Hernández-Sierra JF, Pozos-Guillén Ade J (2007). Comparison of three conscious sedation regimens for pediatric dental patients. J Clin Pediatr Dent.

[22] Lee YJ, Kim do K, Kwak YH, Kim HB, Park JH, Jung JH (2012). Analysis of the appropriate age and weight for pediatric patient sedation for magnetic resonance imaging. Am J Emerg Med.

[23] Kannikeswaran N, Sethuraman U, Sivaswamy L, Chen X, Mahajan PV (2012). Children with and without developmental disabilities: sedation medication requirements and adverse events related to sedation. Pediatr Emerg Care.

[24] Fávero ML, Ponce FA, Pio MR, Tabith Junior A, Carvalho e Silva FL (2010). Chloral hydrate to study auditory brainstem response. Braz J Otorhinolaryngol.

[25] Heistein LC, Ramaciotti C, Scott WA, Coursey M, Sheeran PW, Lemler MS (2006). Chloral hydrate sedation for pediatric echocardiography: physiologic responses, adverse events, and risk factors. Pediatrics.

